# Current perspectives on bioceramic sealers in endodontics: A review

**DOI:** 10.6026/973206300214599

**Published:** 2025-12-15

**Authors:** Lakshmi Durga Alekhya Donipudi, Farzana Shereen, Venkata Soundarya Kolaparthi, Sana Fadlalla, Pranitha Presingu, Huma Tahir

**Affiliations:** 1 Department of Dental surgery, GSL Dental College & Hospital, Rajamahendravaram, Andhra Pradesh, India; 2 Department of Dental Surgery, Kamineni Institute of Dental Sciences, Narketpally, Nalgonda District, Telangana, India; 3 Department of Public Health, Nova Southeastern University, Fort Lauderdale, FL, USA; 4 Department of Dental Surgery, University of Khartoum, Faculty of Dentistry, Khartoum, Sudan; 5 Department of Health Informatics, Grand Valley State University, Allendale, MI, USA; 6 Department of Dental Surgery, University College of Medicine and Dentistry, Lahore, Pakistan

**Keywords:** Bioceramic sealers, Calcium silicate, endodontics, regenerative dentistry, nanotechnology, Zirconia and sealing ability

## Abstract

Bioceramic sealers, particularly calcium silicate-based formulations, have transformed endodontic therapy through superior biocompatibility, sealing and regenerative potential. Their chemical and mechanical interaction with dentin enhances long-term treatment outcomes. Therefore, it is of interest to summarize recent developments in their composition, mechanisms of action, classification, clinical applications and retreatment challenges, noting limitations such as retrievability, handling and cost. Emerging innovations, including nanotechnology and enhanced material rheology, may enhance clinical performance, with long-term clinical studies needed to validate their role in regenerative endodontics.

## Background:

Bioceramics represent a class of biomaterials recognized for their exceptional biocompatibility, making them valuable in both medical and dental fields. Technological and procedural innovations in recent years have significantly enhanced the ability of dental professionals to achieve optimal outcomes in endodontic treatments. A key driver of this advancement is the ongoing development of endodontic materials. Among these, the growing application of bioceramic technology marks a significant shift, signaling a new era in the evolution of endodontic practice [1]. The term "bioceramic" is becoming more prevalent in scientific publications. In the realm of endodontics, it refers to a category of materials primarily based on calcium silicate cements utilized in clinical settings as endodontic sealers. Nonetheless, not all "bioceramic" endodontic sealers possess identical characteristics. Variations in their composition lead to differences in certain properties that are often obscured by the broader label of "bioceramic." The label "bioceramic" was initially applied to BioAggregate® (Innovative BioCeramix, Canada). Given that ceramics are inherently non-metallic inorganic substances, the term "ceramics" encompasses nearly all powders used, including MTA, zinc oxide eugenol (ZOE), glass ionomer (GIC) and zinc oxide-phosphate cements [2]. Bioceramics refer to ceramic materials or metal oxides that are compatible with biological systems, such as alumina, zirconia, bioactive glass, glass ceramics, hydroxyapatite, calcium silicate and resorbable calcium phosphate. In the field of endodontics, bioceramics are predominantly bioactive, with calcium silicate-based cements (CSCs) being the most widely used [3]. Therefore, it is of interest to describe bioceramic sealers in endodontics.

## Bio ceramics:

[1] Bioinert-Alumina, Silica

[2] Bioactive-Calcium Silicate, Bioactive glass, Bioactive glass ceramic, Hydroxyapatite.

[3] Biodegradable-Calcium phosphate, Bioactive glass.

## Bio ceramic products used in Endodontics:

## Putty form:

[1] Proroot MTA

[2] MTA Angelus

[3] NeoMTA Plus

[4] Biodentine

[5] BC Putty

[6] Bioaggregate

[7] CEM Cement

[8] TheraCal LC and others

## Paste form:

[1] BC Sealer

[2] MTA Fillapex

[3] Tech BioSealer

[4] Endoseal MTA and others

## Evolution of bio ceramic sealers:

In the past thirty years, there has been significant interest in creating bioactive dental materials capable of interacting with surrounding tissues and promoting regeneration. Mineral trioxide aggregate (MTA), the first bioactive ceramic utilized in endodontics, is the most extensively researched bioceramic to date. This material was first introduced in the dental field as a root-end filling agent in 1993 and received approval from the Food and Drug Administration (FDA) in 1997. During the early 2000s, numerous modifications of MTA products emerged, successfully addressing the limitations of traditional MTA while preserving its superior performance. During the late 2000s and early 2010s, there was an increase in the development and application of bioceramics in endodontic therapy, which exhibit biological properties that are similar to those of MTA, including antibacterial effects, minimal cytotoxicity and a mild inflammatory response. Clinical practice has extensively utilized products such as Biodentine, EndoSequence Root Repair Material (ERRM), BioAggregate and calcium-enriched mixtures (CEM) [3]. MTA was once considered the gold standard among bioceramic materials, but continuous innovations have aimed to overcome its limitations and enhance its performance. Modern bioceramics now serve multiple purposes across both endodontic and restorative dental procedures. Therefore, keeping current with these developments is vital for making informed choices tailored to specific clinical needs [4].

## Mechanism of action of bioceramic sealers:

Although the exact mechanism by which bioceramic sealers bond to root dentin is not fully understood, several proposed explanations exist for calcium silicate-based sealers:

[1] Structures resembling tags develop alongside an interfacial layer referred to as the "mineral infiltration zone," where alkaline by-products resulting from the hydration of calcium silicate cement degrade the collagen in the surrounding dentin. This breakdown creates a porous network that allows the diffusion of high levels of calcium (Ca^2+^), hydroxide (OH^-^) and carbonate (CO_3_^2-^) ions, promoting enhanced mineralization in that area [5].

[2] When dentin moisture is present, the interaction between calcium and phosphate results in the formation of hydroxyapatite crystals, which occurs alongside the previously established mineral infiltration zone [6].

[3] Bioceramics generate porous powders made up of nanocrystals that range from 1 to 3 nanometers in diameter, effectively inhibiting the adhesion of bacteria. Additionally, their high alkaline pH contributes to their antibacterial properties [7].

[4] They possess inherent osteoinductive potential because they can take in osteoinductive materials when there is a bone healing process in proximity [8].

[5] Act as a regenerative framework of biodegradable lattices that offer a structure that gradually dissolves as the body repairs tissue. The capability to create a superior airtight seal, establish a chemical connection with the tooth structure and possess good radiopacity [8].

## Classification of bio ceramic sealers:

Bio-ceramics are one of the newer materials utilized in endodontics, significantly transforming endodontic treatments. These ceramic substances or metal oxides are biocompatible and feature enhanced sealing properties, as well as antibacterial and antifungal characteristics, rendering them valuable for use in both medical and dental fields. They can either emulate human tissues or break down and promote the healing of natural tissues. This category encompasses substances such as alumina and zirconia, bioactive glass, glass-ceramics, calcium silicates, hydroxyapatite, resorbable calcium phosphates, along with radiotherapy glasses [6]. As shown in Table 1 (see PDF), bioceramic sealers were categorized based on their composition into natural and synthetic types. In natural sealers, the powder is obtained by processing Portland cement, whereas in synthetic sealers, the powder is obtained by laboratory synthesis of calcium silicate. We further divided them according to the form of material supply-premixed and non-pre-mixed [9].

## Other classifications include [4]:

[1] Bioinert: Sealers those are non-interactive with biological systems, such as Alumina and Zirconia.

[2] Bioactive: Sealers that undergo interfacial interactions with the surrounding tissue, such as Bioactive glass ceramics and calcium silicates.

[3] Biodegradable: Sealers those are soluble or resorbable and eventually replaced by tissue, such as Tricalcium phosphate and bioactive glasses.

## Key properties relevant to clinical success:

Bioceramic root canal sealers have emerged as a noteworthy innovation in endodontics, gaining attention for their unique properties and clinical advantages over traditional sealers [10]. These materials are evaluated using Grossman's ideal sealer criteria, which emphasize properties such as adequate tackiness for adhesion, hermetic sealing ability, radiopacity, small particle size for application ease, dimensional stability without shrinkage, no tooth discoloration, bacteriostatic behavior, delayed setting time, resistance to dissolution in tissue fluids, biocompatibility and retrievability using common solvents [11]. A defining characteristic of bioceramic sealers is their excellent biocompatibility. As calcium silicate-based materials, they are well tolerated by periapical tissues and can support healing even in cases of extrusion beyond the apex [12]. Moreover, their bioactivity and the ability to induce hydroxyapatite formation upon interaction with surrounding tissues further enhance sealing and contribute to periapical tissue regeneration [13]. Unlike traditional resin-based materials, bioceramic sealers exhibit slight expansion upon setting, improving adaptability and contributing to a superior hermetic seal that enhances long-term success. Their high alkaline pH during the setting phase creates an antimicrobial environment within the canal system, aiding in the reduction of residual bacterial load [7]. Clinically, these sealers are sufficiently radiopaque, allowing for clear visualization during post-obturation assessment. Recent improvements, such as pre-mixed, syringe-based formulations, have streamlined handling and reduced technique sensitivity, improving clinical efficiency [12]. One limitation discussed in the literature is their challenging retrievability. The strong adhesion to dentin walls and chemical stability of set bioceramic sealers can complicate removal during retreatment procedures [9]. In summary, bioceramic root canal sealers demonstrate a strong alignment with Grossman's ideal properties. Their biocompatibility, bioactivity, dimensional stability, antimicrobial potential and improved handling characteristics make them a valuable asset in endodontic therapy, with considerable promise for enhancing long-term clinical outcomes [14].

## Clinical applications:

Bioceramic materials are widely used in endodontics and are available in various forms, such as sealers, pastes and putties. Putties are commonly used in procedures like root-end filling, vital pulp therapy, apexification, regenerative endodontic therapy, perforation repair and repair of root defects. Pastes, such as BioRoot RCS and BC Sealer, are mainly used as sealing agents during root canal treatments. These materials have gained popularity because of their excellent physical and biological properties, which make them easier to use, even for clinicians with limited experience [1]. Bioceramic sealers are available in two forms: a powder-liquid formulation that requires manual mixing, making it technique-sensitive and potentially leading to inconsistent handling and a premixed formulation that sets upon contact with moisture from the surrounding tissues. Premixed sealers offer the advantage of consistent texture and improved ease of handling during clinical procedures by reducing the chances of error [11]. The advantages of bioceramic materials used as root canal sealers are their excellent biocompatibility, lower cytotoxicity, higher level of cell attachment and the presence of calcium phosphate, which helps strengthen the bond between the sealer and root dentin. Some studies also suggest that when these sealers are accidentally pushed beyond the root tip (apical foramen) during filling or perforation repair, they may support bone regeneration in the surrounding area. Some *in vitro* studies have shown that bioceramic materials can improve the fracture resistance of teeth that have undergone root canal treatment, especially when used with gutta-percha cones that are coated or impregnated with bioceramic [10]. Over the years, many types of endodontic sealers have been developed (such as iRoot SP, EndoSequence BC, Totalfill BC, etc), each with its unique features, benefits and drawbacks. The selection of a sealer often depends on factors such as the clinician's experience, preference and the specific properties of the material [15]. Several obturation techniques are used with sealers, including the single-cone technique, cold lateral condensation, warm vertical compaction and carrier-based methods. The main goal of these methods is to enhance the quantity of gutta-percha utilized while minimizing the amount of sealer used, resulting in a more secure and long-lasting seal. Among them, the single-cone technique is the easiest and most straightforward to perform [13]. Recent research indicates that applying heat during obturation can significantly affect the physical and chemical properties of bioceramic sealers. However, the extent of these changes varies depending on the specific composition of the sealer, the temperature used and the duration of heat exposure. Some bioceramic sealers remain stable even under heated conditions [14]. Despite these variations, warm obturation techniques have their advantages. For instance, a study by Dasari *et al.* found that the warm vertical compaction technique achieved the greatest depth of sealer penetration into the dentinal tubules, followed by the injectable gutta-percha technique. The sealer's viscosity and flowability mainly influence the depth of penetration. Effective penetration and strong marginal sealing are crucial for the long-term success of endodontic treatment, as they help prevent microleakage and bacterial reinfection across diverse clinical environments [15].

## Challenges and Limitations:

Calcium silicate-based bioceramic sealers, such as EndoSequence BC Sealer, TotalFill BC and BioRoot RCS, have gained prominence in endodontics due to their exceptional biocompatibility, bioactivity and sealing properties. Their hydrophilic nature and ability to form hydroxyapatite in situ promote superior sealing and periapical healing. However, their advantageous physical and chemical interactions with dentinal structures present substantial challenges during non-surgical endodontic retreatment (NSER) [16]. Material-Dentin Interface and Retention: Bioceramic sealers form a mineral infiltration zone within the dentin, accompanied by the deposition of hydroxyapatite-like crystals. This results in micromechanical interlocking and chemical bonding to dentinal tubules, complicating their complete removal during retreatment [17]. Once set, these materials become extremely hard and are resistant to removal using conventional files or endodontic solvents. Their high affinity for dentinal walls leads to persistent remnants even after aggressive instrumentation [16]. Retrievability and removal technique: Studies consistently show that no single technique can completely remove bioceramic sealers. In a comparative study, Crozeta *et al.* reported that while rotary instrumentation was more effective than hand files, it still failed to achieve complete debridement, especially in the apical third [17]. Ultrasonics and photon-induced photoacoustic streaming (PIPS) techniques improved retrieval in straight canals, but residual material persisted in complex anatomies. Moreover, commonly used solvents such as chloroform or eucalyptol, effective against gutta-percha and resin-based sealers, do not significantly soften bioceramics [18]. The obturation method also influences retreatability. A systematic micro-CT review noted that warm vertical compaction alters the physical properties of bioceramic sealers, increasing hardness and hindering subsequent removal compared to cold lateral compaction. Heat may degrade the crystalline matrix or cause expansion, further embedding the material within the canal walls [19]. Instrumentation Force and Torque: Mechanical retreatment in bioceramic-filled canals generates different stress profiles compared to traditional sealers. An *in vitro* study using XP-endo Shaper revealed that while apical forces were lower, significant remnants remained, particularly in the apical third [20]. Torque and coronal forces were comparable to those seen with AH Plus sealer retreatments, but bioceramic sealers required longer instrumentation time and additional activation methods to reach acceptable cleanliness [21].

## Re-establishing working length and apical patency:

Bioceramic sealers pose a risk to re-establishing apical patency and working length due to their tenacious sealing. BioRoot RCS, in particular, has been linked to a higher frequency of failure in regaining apical patency compared to epoxy-based AH Plus [21]. However, novel sealers like Neosealer Flo, when combined with advanced rotary systems, have demonstrated improved patency rates *in vitro*, indicating that sealer formulation and retreatment tools are critical determinants [20].

## Chemical adjuncts and clinical limitations:

Adjunctive use of formic acid (10%) has shown promising results in dissolving set bioceramic sealers and restoring canal patency, outperforming traditional solvents. Nevertheless, its clinical adoption remains limited due to concerns regarding safety and standardization [22]. Clinically, retreatment difficulty is pronounced due to the hardness and insolubility of bioceramics once set. They often require the use of aggressive ultrasonics or drilling, which increases the risk of iatrogenic damage [18]. Their unpredictable setting behavior, especially in moist environments or when using premixed formulations, can lead to uneven curing and complicate future intervention. Studies show persistent remnants despite advanced removal techniques [19].

## Clinical recommendations and future directions:

Bioceramic sealers offer a promising future in endodontics due to their biological compatibility, sealing ability and regenerative properties. However, their performance can be improved through continued material development [23]. Incorporating nanomaterials such as silver nanoparticles has demonstrated enhanced antimicrobial activity and improved mechanical properties without compromising physical integrity [24]. Despite these advances, challenges persist in handling. Material consistency, flow and retrievability during retreatment remain concerns that require innovation to ensure ease of use and predictable outcomes [25]. Additionally, although many formulations have shown favorable results in the short term, there is a lack of long-term clinical evidence evaluating outcomes such as dimensional stability, periapical healing and interaction with host tissues [26]. Cost is another important consideration. Bioceramic sealers are often expensive and less accessible in lower-income regions, limiting their widespread adoption. Future work should prioritize affordable, scalable formulations that retain their biological and physical performance [24, 26]. These sealers are also well-suited for simplified obturation approaches, such as the single-cone technique, which reduces operator variability and supports efficient workflows. Beyond root canal obturation, their application is expanding into treatments such as specification; pulp capping and perforation repair, highlighting their role in regenerative therapies [23, 25]. Moving forward, clinical research should focus on multicenter trials, standardized evaluation metrics and extended follow-up periods to fully establish the clinical value and safety of these materials.

## Conclusion:

With advances in endodontic materials, bioceramic sealers have emerged as key innovations that integrate biological sciences with material science to redefine root canal therapy. Bioceramic materials urge clinicians to rethink traditional protocols and embrace biologically driven, minimally invasive, patient-centered approaches. As the field moves forward, bioceramic sealers are poised not only to replace older materials but also to redefine expectations for what endodontic outcomes can achieve.
